# Knowledge, Attitudes, and Practices about Hyperuricemia and Gout in Community Health Workers and Patients with Diabetes

**DOI:** 10.3390/healthcare12111072

**Published:** 2024-05-24

**Authors:** Shiyi Sun, Lihong Chen, Dawei Chen, Yan Li, Lin Ma, Yumin Hou, Yuhong Liu, Xingwu Ran

**Affiliations:** 1 Department of Endocrinology & Metabolism, West China Hospital, Sichuan University, Chengdu 610041, China; sunshiyi2021@163.com (S.S.); chenlihong@scu.edu.cn (L.C.); wdc104@163.com (D.C.); 2 Innovation Research Center for Diabetic Foot, Diabetic Foot Care Center, West China Hospital, Sichuan University, Chengdu 610041, China; 3 Department of Clinical Research Management, West China Hospital, Sichuan University, Chengdu 610041, China; liyan0217@wchscu.cn; 4 West China School of Nursing, Sichuan University, Chengdu 610041, China; malintulip@163.com; 5 Wannian Community Health Center in Chenghua District, Chengdu 610051, China; houyumin0427@163.com; 6 The Third People’s Hospital of Chenghua District, Chengdu 610051, China; lyh6917@163.com

**Keywords:** diabetes, hyperuricemia, gout, questionnaire, knowledge

## Abstract

Hyperuricemia exhibits a high incidence among individuals with diabetes; however, the significance of hyperuricemia and gout is often underestimated. This study aimed to assess the knowledge, attitude, and practice of hyperuricemia and gout among community health workers and patients with diabetes. Two questionnaires were designed to investigate knowledge, attitudes, and practices of hyperuricemia and gout among community health workers and patients with diabetes in Chenghua District, Chengdu, from August 2021 to January 2022. A total of 709 community health workers were included, whose average score was 17.74/30. Approximately half of general practitioners (GPs) demonstrated knowledge regarding the target serum uric acid levels for hyperuricemia and gout. Only 11.2% of GPs were fully aware of the preferred medicine for acute gout. The majority of GPs (86.7%) demonstrated limited awareness regarding the contraindications associated with colchicine, while a significant proportion (65.1%) lacked knowledge about the specific classes of drugs that inhibit uric acid synthesis. Among the 508 patients with diabetes included in this survey, 32.3% demonstrated awareness of hyperuricemia, while 60.8% exhibited knowledge regarding gout. The average score attained by these individuals was recorded at 7.21 out of a total of 26 points. The majority of patients with diabetes (87.8%) held the mistaken belief that hyperuricemia definitely led to the development of gout. Almost 66% agreed that a massage or a hot compress could be used when acute gouty arthritis attacks. The knowledge rate of hyperuricemia and gout among community health workers was moderate, while it was low in patients with diabetes.

## 1. Introduction

Hyperuricemia is a chronic metabolic disease with abnormally elevated serum uric acid (SUA) levels, characterized by increased production and/or decreased excretion of uric acid. Several diseases, including cardiovascular disease, hypertension, diabetes, and renal injury, are often related to hyperuricemia [1]. Hyperuricemia is considered to be the prestage of gout, and all individuals with gout exhibit an accumulation and crystallization of uric acid [2]. Gout often causes restrictions on joint movements, joint deformities, and difficulty fitting shoes. Moreover, ulcers and superimposed infections are also gout complications [3].

The overall hyperuricemia prevalence among Chinese adults was 11.1% in 2015–2016 and 14.0% in 2018–2019 [4]. Hyperuricemia was higher in men than women in 2015–2016 (19.3% vs. 2.8%) and 2018–2019 (24.4% vs. 3.6%) [4]. Liu et al. [5] revealed that persistent hyperuricemia was associated with a 75% higher risk of diabetes. Diabetes is an independent factor in hyperuricemia. The overall prevalence of hyperuricemia in patients with diabetes was 32.6% (36.1% in women, 28.4% in men), which was significantly higher than that in the general population in South China (18.6%) [6,7]. Moreover, Lai et al. [8] demonstrated that gout and type 2 diabetes shared a set of 36 genes, indicating a mutually interdependent relationship leading to higher incidences. Not all patients with hyperuricemia will develop gout; therefore, gout is a subset of patients with symptomatic hyperuricemia [9]. The recurrence of gout flares is associated with the severity of hyperuricemia. Gout and hyperuricemia share similar risk factors, including some diseases, medications, and environmental exposures. In addition, joint trauma and acute illness are also related to gout flares [3].

The prevalence and prognosis of hyperuricemia and gout are closely related to living habits and adherence to drug therapy [4,10,11]. Compliance with drug treatment among gout patients is poor, with compliance rates of 50% or less [12]. Correct treatment and patient education from doctors as critical regulators of patients’ healthy lifestyles and good compliance are essential to reduce the incidence and improve the prognosis [11,13]. Nurse-led education has shown significant improvement in reaching its goals. Pharmacist-led interventions had likewise succeeded to a lesser degree [13]. Previous studies conducted in Saudi Arabia, Poland, and the Republic of Croatia have revealed a notable lack of physicians’ awareness regarding hyperuricemia, especially concerning drugs, pathophysiology, and risk factors [14,15,16]. Moreover, a considerable number of patients in Southwest China suffering from hyperuricemia remain unaware of their condition [17]. A minority of gout patients were aware of common foods triggering gout and the causes and consequences of gout, and only 12% of those receiving uric acid-lowering treatment knew the short-term risks of worsening gout with initiation [18,19].

Chenghua District, located in Chengdu, Sichuan, China, is characterized by its substantial size and gradual pace of urban development, accompanied by a comparatively lower socioeconomic status. Chenghua District is characterized by a substantial elderly population afflicted with a range of chronic ailments. The management of hyperuricemia or gout necessitates a protracted therapeutic course; however, the administration of urate-lowering therapy may be constrained by adverse effects and severe hypersensitivity reactions [20]. In addition to drugs, lifestyle intervention is also significant for the treatment of hyperuricemia or gout [21,22]. Letting patients fully understand the relevant knowledge regarding hyperuricemia or gout can encourage patients to promote healthy lifestyles and undergo early screening, diagnosis, and treatment.

Because of the considerably high prevalence (21.24%) of hyperuricemia observed in our previous study conducted in Chenghua District among patients with diabetes [23], and because limited research has been conducted on the knowledge of hyperuricemia and gout among individuals with diabetes and healthcare professionals, we conducted cross-sectional surveys in Chenghua District to explore the knowledge, attitudes, and practices pertaining to hyperuricemia and gout among community health workers, and we also investigated the knowledge of patients with diabetes. This study would provide valuable insights into the gaps in training for community health workers as well as deficiencies in health education for patients.

## 2. Methods

### 2.1. Questionnaire Development

Two questionnaires for community health workers and patients with diabetes were developed based on the Guidelines for Primary Care of Gout and Hyperuricemia: Practice Version (2019) and the Guidelines for the Diagnosis and Management of Hyperuricemia and Gout in China (2019) [24,25]. Before use, a pilot survey for community health workers was conducted on 30 individuals, and the two questionnaires were subsequently reviewed and adjusted by a panel of five endocrinological experts to ensure the validity of their structure and content.

The final questionnaire for community health workers comprised three dimensions: demographic information (occupations, ages, genders, and educational levels), a section on practice and attitude, and a knowledge part that primarily encompassed pathophysiology (4 items), drug treatments (16 items), lifestyle interventions (8 items), and harmful effects (2 items). The types of questions included single-choice, multiple-choice, and judgment questions. The participant was given a score of 1 if they answered the question correctly, ranging from 0 to 30, and each question must be answered. Moreover, 0–10 points were considered low knowledge rates, 11–20 points were defined as moderate knowledge rates, and 21–30 points were regarded as high knowledge rates (Supplementary Materials).

The questionnaire for patients with diabetes encompassed demographic information (ages, genders, educational levels, and history of hyperuricemia or gout), a section on practices and attitudes, as well as a knowledge component comprising pathophysiology (5 items), treatments (16 items), and harmful effects (5 items). All questions were framed as judgment questions, requiring participants to determine whether the statements were true, false, or unclear. Participants who possessed knowledge about hyperuricemia or gout could answer the relevant items accurately; otherwise, they received a score of 0 for that particular section. A score of 1 was assigned if participants responded correctly to a question, resulting in a range from 0 to 26 points. Low knowledge rates were defined as scores between 0 and 9 points; moderate knowledge rates ranged from 10 to 18 points; and high knowledge rates encompassed scores between 19 and 26 points (Supplementary Materials). The electronic questionnaire was administered through an application (https://www.wenjuan.com).

### 2.2. Population and Data Collection

The cross-sectional survey for community health workers, including general practitioners (GPs), nurses and others (health administrators, pharmacists, laboratory examiners, toll room workers, radiologists, psychotherapists and medical technicians), was conducted in all primary hospitals (the Third People’s Hospital, the Seventh People’s Hospital, the Sixth People’s Hospital, respectively, Wannian Community Health Center, Tiaodeng River Community Health Center, Longtan Community Health Center, Shuangqiaozi Community Health Center, Erxian Bridge Community Health Center, Fuqing Road Community Health Center, Baohe Community Health Center and Qinglong Community Health Center, respectively) in Chenghua District, Chengdu from August 2021 to September 2021. The QR code was distributed to the official WeChat group of primary hospitals in Chenghua District, inviting all community health workers to participate in an online questionnaire by scanning the QR codes using their mobile devices. One account could only fill out a questionnaire once. All questions must be fully answered before submission. We promptly contacted individuals who failed to complete the questionnaire via telephone until all community health workers successfully participated in the survey.

We employed simple random sampling to select four primary hospitals from a pool of eleven in Chenghua District. Another cross-sectional survey was performed from November 2021 to January 2022 on patients with diabetes in the four primary hospitals (the Third People’s Hospital, Wannian Community Health Center, Tiaodeng River Community Health Center, and Baohe Community Health Center). The sample size of 402 was calculated using the confidence intervals for one proportion in the PASS 11 software based on the assumption that the knowledge rate of hyperuricemia or gout was 50% with a two-sided 95% confidence interval and a width equal to 0.100. To prevent data unavailability, we increased the sample size by approximately 20% on top of 402. Convenience sampling was employed in this study to recruit individuals diagnosed with diabetes, and a trained professional endocrinologist collected the questionnaire through a face-to-face interview and synced it on the web by smartphone. All questions must be fully answered before submission. Patients who met the 1999 WHO diagnostic criteria for diabetes and were willing to participate in the study were enrolled. Patients with mental disorders and hearing or speaking problems were excluded. All questionnaire results should be obtained by logging into a specific account and password. The study was registered at the Chinese Clinical Trial Registry (27 January 2021, ChiCTR2100042742).

### 2.3. Statistical Analysis

SPSS 23 (SPSS Company, Chicago, IL, USA) was used for statistical analysis. Numbers (percentages) and means (standard deviations) were used for statistical description. Differences among groups were analyzed by the chi-squared test (categorical variables), Mann–Whitney U-test (non-normal data), and Kruskal–Wallis test (non-normal data and ordinal categorical variables). Multiple linear regression was used to assess the association between the knowledge scores and sociodemographic factors. A *p*-value of ≤0.05 was considered statistically significant.

## 3. Results

### 3.1. Characteristics of Community Health Workers

A total of 709 community health workers, ranging in age from 20–78 years, were included in this survey conducted across all primary hospitals, resulting in a remarkable response rate of 100%. As shown in Table 1, most community health workers were female, with junior college degrees constituting 34.98% and bachelor’s degrees constituting 59.80%. The population included 278 GPs, 319 nurses, and 112 other community health workers.

### 3.2. Practices and Attitudes of Community Health Workers for Patient Education

**Figure 1:**
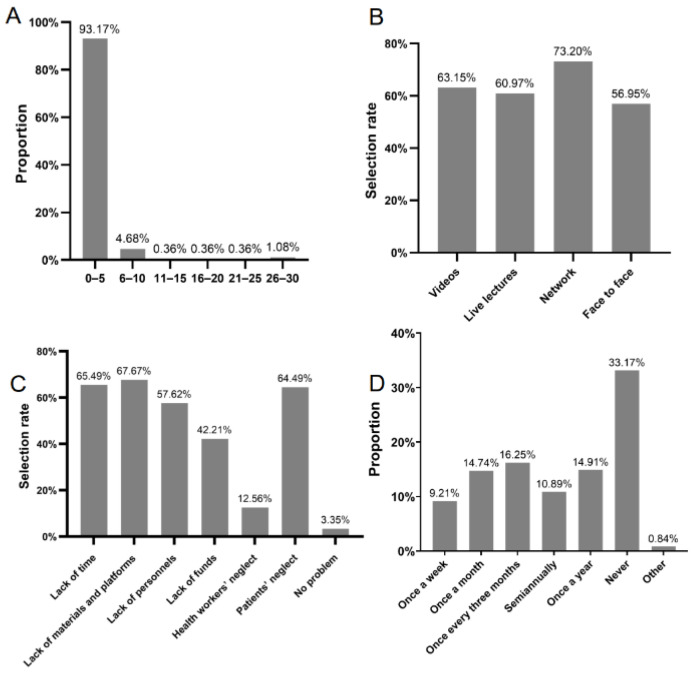
Investigation results of patient education among community health workers. (**A**) Average number of hyperuricemia or gout patients treated by GPs at each outpatient work; (**B**) education methods preferred by GPs and nurses; (**C**) reasons for patient education difficulties; (**D**) frequency of GPs’ and nurses’ propaganda on hyperuricemia and gout.

Approximately 93.2% of GPs treated between zero and five patients with hyperuricemia or gout during each outpatient session (Figure 1A). GPs and nurses preferred educating patients through videos (63.15%), live lectures (60.97%), and networks (73.20%) (Figure 1B). Lack of time (65.49%), materials and platforms (67.67%), personnel (57.62%), and patients’ neglect (64.49%) hindered patient education (Figure 1C). A total of 33.17% of GPs (20.44%) and nurses (12.73%) had never conducted patient education on hyperuricemia and gout, and only 9.21% of GPs and nurses were able to conduct patient education once a week (Figure 1D).

### 3.3. Characteristics of Patients with Diabetes

**Figure 2:**
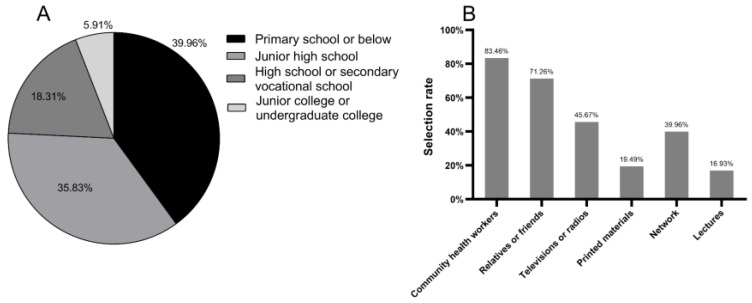
Demographic information of patients with diabetes. (**A**) Educational levels; (**B**) sources of health knowledge.

A total of 513 diabetic patients were included in our investigation, with a response rate of 99.03%. Among them, five patients declined participation due to time constraints, resulting in a final sample size of 508 participants who completed the study. The age of the included patients with diabetes was 65.49 ± 9.41 years (range 34–86 years), 47.83% of whom were male. Their educational levels were generally low, with junior high school and primary school or below accounting for 35.83% and 39.96%, respectively (Figure 2A). Of the participants, 32.48% had previously suffered from hyperuricemia, and 5.12% did not know whether they had ever suffered from hyperuricemia. In addition, 9.84% of patients with diabetes had been diagnosed with gout in the hospital, and 3.94% of patients with diabetes had similar symptoms but were not diagnosed. 

### 3.4. Practices and Attitudes of Patients with Diabetes for Education

The respondents acquired health knowledge mainly from community health workers (83.46%) and relatives or friends (71.26%) (Figure 2B). Televisions or radios (45.67%) and networks (39.96%) were the main ways for the elderly and young people to obtain health knowledge, respectively. Of the participants, 62.60% (including 88 people who had suffered from hyperuricemia or gout) were unwilling to receive training on hyperuricemia and gout. The main reasons included that they lacked time, did not suffer from hyperuricemia or gout, or did not pay attention to hyperuricemia or gout. Approximately 37.40% of respondents were willing to participate in the training, among which 54.21% had not suffered from hyperuricemia or gout, and they wanted to accept more health knowledge.

### 3.5. Knowledge of Hyperuricemia and Gout among Community Health Workers

The average score of this survey was 17.74/30, without significant differences between genders. Community health workers aged under 30 years had the lowest scores, and the average score increased gradually with the improvement of educational levels. In addition, GPs scored the highest, statistically significant, compared with nurses and others (Table 2).

Correct responses regarding pathophysiology were provided by GPs (60.25%) more frequently than nurses (42.24%) and other community health workers (45.31%). GPs had poor knowledge of the stages of hyperuricemia. Most nurses and other community health workers knew that gout patients must have hyperuricemia at a particular stage (93.10% and 92.86%), whereas the correct rates of the remaining items were low (Supplementary Materials Table S1).

Regarding drug treatments, the average correct rates of GPs, community health workers, and nurses were 60.32%, 49.11%, and 46.43%, respectively. Notably, a substantial proportion of GPs (80.22%) held the mistaken belief that if gout occurs once annually, it must start with a reduction in uric acid levels. In addition, most GPs were unaware of target SUA levels (55.04%), the preferred drugs for acute gout attack (88.85%), contraindications to colchicine (86.69%), and drugs inhibiting uric acid synthesis (65.11%), but the correct rates of other items were more than 50%. (Supplementary Materials Table S1).

In the lifestyle interventions section, the average correct rate of GPs was still the highest (73.87%), and the average correct rate of nurses (64.65%) was slightly higher than that of other community health workers (63.73%). Most GPs (88.49%), nurses (97.18%), and other community health workers (99.11%) were puzzled about weight management and regular exercise requirements. Moreover, only 34.48% of nurses and 45.54% of other community health workers answered questions about red meat types correctly (Supplementary Materials Table S1).

The majority of community health workers (>90%) demonstrated accurate knowledge regarding the detrimental effects of hyperuricemia and gout (Supplementary Materials Table S1).

### 3.6. Knowledge of Hyperuricemia and Gout among Patients with Diabetes

Among the 508 patients diagnosed with diabetes, a notable proportion of 32.28% demonstrated awareness regarding hyperuricemia, while a substantial majority of 60.83% exhibited knowledge about gout; furthermore, the average score for their overall understanding was recorded as 7.21 out of a total possible score of 26. Hence, the knowledge rate of gout was significantly higher than that of hyperuricemia. Approximately 31.5% of participants knew of hyperuricemia and gout, whereas 38.39% knew neither hyperuricemia nor gout. Among the patients with diabetes suffering from hyperuricemia, 50% knew of hyperuricemia. In addition, among patients with diabetes suffering from gout, 92% knew of gout. Significant variations in knowledge rates and scores were observed among different genders, age groups, education levels, and individuals with a history of hyperuricemia or gout (Table 3).

The average correct rate of pathophysiology was 43.94%. Except for the question that uric acid was mainly excreted from urine, the proper rates of other questions were below 50%. Most patients with diabetes mistakenly believed that the diagnosis of hyperuricemia primarily depended on urine examination (62.80%) and were unaware that hyperuricemia and gout were hereditary (70.12%) (Supplementary Materials Table S2). The average accuracy rate for identifying harmful effects was 61.34%. The vast majority of patients with diabetes (96.95%) reported that hyperuricemia was related to gout, but most of them (87.80%) erroneously considered that hyperuricemia would definitely develop gout (Supplementary Materials Table S2). Finally, the average correct response rate of the treatments was the highest (72.70%). Nevertheless, most patients with diabetes erroneously deemed that hyperuricemia must be treated with drugs (71.34%), and a massage and a hot compress could be used when acute gouty arthritis occurred (66.67%) (Supplementary Materials Table S2).

### 3.7. Factors Influencing Knowledge Scores

In the multivariable analysis, females and GPs were associated with higher knowledge scores among community health workers. However, ages and educational levels did not significantly influence knowledge scores (Supplementary Materials Table S3). In patients with diabetes, higher levels of education and a history of hyperuricemia or gout were found to be positively associated with greater knowledge scores regarding hyperuricemia or gout (Supplementary Materials Table S4).

## 4. Discussion

The community health workers’ knowledge rate of hyperuricemia and gout was moderate. In addition, the knowledge rate of hyperuricemia and gout was low in patients with diabetes, and half of the patients with hyperuricemia did not know about hyperuricemia. Compared with hyperuricemia, patients with diabetes had a higher knowledge rate of gout. Among the patients with diabetes who knew about hyperuricemia or gout, their knowledge rates of pathophysiology and treatments were insufficient.

Although primary health workers’ perceptions of gout and hyperuricemia-related knowledge varied in different areas, they had certain similarities in certain parts. Zuzic et al. [14] revealed that approximately half of the primary care physicians correctly identified drugs that could lower or elevate SUA, and primary care physicians poorly understood the pathophysiology and risk factors of hyperuricemia. The correct rate of pathophysiology in our study was also low (49.79%). Alqarni et al. [15] showed that only 32.8% of participating Saudi primary healthcare physicians had adequate knowledge about asymptomatic hyperuricemia, which increased the risk of misprescription, including anti-inflammatory medications and urate-lowering therapy. Spaetgens et al. [26] revealed that GPs lacked familiarity with target-level SUA (12.5%) and knowledge about the duration of uric acid-lowering therapy and adequate prophylactic treatment. Similarly, our research also revealed that approximately half of GPs knew the target level of SUA, and less than 20% knew when to start uric acid-lowering therapy for gout sufferers. A study already showed inappropriate use of prophylactic colchicine among 74% of the patients under primary care [27]. In our survey, only 13.31% of GPs knew the suitable eGFR range for colchicine use. Approximately half of the GPs believed diet and drinking habits were the main risk factors for gout in the Netherlands [26]. In our study, community health workers had a relatively higher correct rate (68.11%) of lifestyle intervention.

Previous papers have shown that poor disease-related knowledge among gout patients is widespread. Zhang et al. [28] discovered poor patient knowledge about optimum SUA levels, compositions inside the joint causing attacks of gout, and the duration of uric acid-lowering drugs. An enlarged survey of southern Chinese gout patients showed that patients lacked knowledge about the cause of gout attacks [29]. A qualitative study of 20 British gout patients revealed that most respondents were not aware of the cause of gout and that gout could be treated effectively by lifestyle change and uric acid-lowering drugs [19]. In an investigation enrolling 60 gout sufferers in New Zealand, 85% agreed that diet could trigger gout attacks, but less than 50% knew how uric acid-lowering drugs worked, and only 33% knew which drugs to use both acutely and chronically [30]. Despite beliefs that gout was caused primarily by diet and alcohol intake, patients in New Zealand did not perceive that gout was strongly influenced by their actions [31]. Our study differed from the previous study in that the subjects were diabetes patients, and our questionnaire did not involve specific uric acid-lowering drugs or target SUA levels. In our study, a majority of respondents who knew about hyperuricemia or gout agreed that hyperuricemia and gout were related to beer and diet.

Patients and community health workers valued the obvious pain and joint deformation caused by gout, while asymptomatic hyperuricemia was often ignored [20]. Thus, a proportion of patients with hyperuricemia remained undiagnosed in the primary healthcare system [17]. Most gout patients did not receive standardized treatments but only took analgesics when they were in pain [20]. Once their symptoms improved, they no longer attached importance to gout, resulting in repeated gout attacks. Similar to our study, research conducted in primary care in the Netherlands showed that only 12.9% had followed an educational event on gout in the past year [26]. GPs lacked attention to adherence to uric acid-lowering therapy and did not give any lifestyle advice to patients [26]. Furthermore, 33.17% of GPs and nurses never conducted relevant education in our study. Patient neglect and a lack of time, materials, and platforms might prevent community health workers from adequately popularizing relevant knowledge.

This study encompassed comprehensive inquiries regarding the pathophysiology, treatment modalities, and harmful effects, with a relatively large sample size. However, this study also had some limitations. Firstly, the community health workers were busy, so the doctor’s tight schedule might influence their responses. Secondly, most patients with diabetes participating in the survey had good compliance, so the usual knowledge rate might be lower. Finally, the questionnaires for this study were determined according to local medical conditions and cultural levels, so they might not thoroughly apply to all primary hospitals.

## 5. Conclusions

The level of knowledge regarding hyperuricemia and gout among community health workers was deemed moderate, whereas it was found to be low in patients with diabetes. It is crucial to design and carry out rigorous continuing medical education programs for community health workers and appropriate patient education for patients with diabetes.

## Figures and Tables

**Table 1:** Demographic information on community health workers.

Variable	Total (n = 709)	GPs (n = 278)	Nurses (n = 319)	Others (n = 112)	*p*-Value
Age (years)	33.93 ± 7.42	36.97 ± 8.10	31.55 ± 5.89	33.19 ± 6.92	<0.001 *
Gender (Male/Female)	96/613	63/215	9/310	24/88	<0.001 *
**Educational Levels**					
Secondary vocational school education	20 (2.82%)	7 (2.52%)	7 (2.19%)	6 (5.36%)	<0.001 *
Junior college degree	248 (34.98%)	55 (19.78%)	158 (49.53%)	35 (31.25%)	
Bachelor’s degree	424 (59.80%)	201 (72.30%)	154 (48.28%)	69 (61.61%)	
Master’s degree	17 (2.40%)	15 (5.40%)	0 (0.00%)	2 (1.79%)	

Abbreviation: GPs, general practitioners; * *p* ≤ 0.05.

**Table 2:** Knowledge scores of community health workers.

Variable	Numbers	Knowledge Scores	*p*-Value
Total	709	17.74 ± 3.48	-
**Gender**			
Male	96	17.53 ± 3.32	0.953
Female	613	17.78 ± 3.51	
**Age (Years)**			
<30	194	16.68 ± 3.10	<0.001 *
30–39	387	18.06 ± 3.66	
40–49	101	18.50 ± 3.21	
≥50	27	18.04 ± 2.84	
**Educational Levels**			
Secondary vocational school education	20	16.25 ± 2.07	<0.001 *
Junior college degree	248	16.98 ± 3.25	
Bachelor’s degree	424	18.17 ± 3.52	
Master’s degree	17	19.94 ± 4.31	
**Occupations**			
GPs	278	19.89 ± 3.26	<0.001 *
Nurses	319	16.24 ± 2.71	
Others	112	16.71 ± 3.24	

Abbreviation: GPs, general practitioners; * *p* ≤ 0.05.

**Table 3:** Knowledge rates and scores of hyperuricemia and gout among patients with diabetes.

Variable	Numbers	Know about Hyperuricemia or Gout n (%)	*p*-Value	Knowledge Scores	*p*-Value
Total	508	313 (61.61%)	-	7.21 ± 7.64	-
**Gender**					
Male	243	174 (71.60%)	<0.001 *	8.23 ± 7.59	<0.001 *
Female	265	139 (52.45%)		6.28 ± 7.57	
**Age (Years)**					
<50	23	20 (86.96%)	<0.001 *	11.96 ± 7.57	<0.001 *
50–69	297	201 (67.68%)		7.75 ± 7.62	
≥70	188	92 (48.94%)		5.78 ± 7.36	
**Educational Levels**					
Primary school or below	203	81 (39.90%)	<0.001 *	4.29 ± 6.67	<0.001 *
Junior high school	182	131 (71.98%)		8.56 ± 7.40	
High school or secondary vocational school	93	74 (79.57%)		8.97 ± 7.71	
Junior college or undergraduate college	30	27 (90.00%)		13.33 ± 7.70	
**History of Hyperuricemia or Gout**					
Yes	165	122 (73.94%)	<0.001 *	10.17 ± 8.20	<0.001 *
No	343	191 (55.69%)		5.79 ± 6.92	

* *p* ≤ 0.05.

## Data Availability

The raw data supporting the conclusions of this article will be made available by the authors on request.

## Supplementary Materials

The following supporting information can be downloaded at: https://www.mdpi.com/article/10.3390/healthcare12111072/s1, Table S1. Knowledge rate of hyperuricemia and gout among community health workers. Table S2. Knowledge rate of hyperuricemia and gout among patients with diabetes. Table S3. Multiple linear regression on factors associated with knowledge scores in community health workers. Table S4. Multiple linear regression on factors associated with knowledge scores in patients with diabetes.
