# Supplementary material for: Knowledge, Attitudes, and Practices about Hyperuricemia and Gout in Community Health Workers and Patients with Diabetes
Source: Healthcare (Basel). 2024 May 24;12(11):1072. doi: 10.3390/healthcare12111072 (PMC11171554; doi:10.3390/healthcare12111072)
Supplement: Supplementary file 1 [file healthcare-12-01072-s001.zip › healthcare-2987804-supplementary.pdf]

Part one: Questionnaire on hyperuricemia and gout among community health workers

Demographic information

1. Which is your occupation?

A Doctors

B Nurses

C Others

2. Which is your gender?

A Male

B Female

3. Age _____years old

4. Which is your highest academic qualification (including currently studying)?

A Secondary vocational school education

B Junior college degree

C Bachelor’s degree

D Master’s degree

E Doctor’s degree or above

F Others

Practices and attitudes

1. The average number of patients with hyperuricemia or gout treated by you during each outpatient session is

A 0–5

B 6–10

C 11–15

D 16–20

E 21–25

F 26–30

2. How often do you conduct patient education on hyperuricemia and gout?

A Once a week

B Once a month

C Once every three months

D Semiannually

E Once a year

F Never

G Others

3. Which of the following ways do you prefer to conduct hyperuricemia and gout education? (multiple choice)

A Videos

B Live lectures

C Networks

D Face-to-face explanation during the patient visit

4. Which of the following factors do you think to make it challenging to teach patients? (multiple choice)

A Lack of time

B Lack of materials and platforms

C Lack of personnels

D Lack of funds

E Health workers' neglect

F Patients' neglect

G No problem

Knowledge

1. How much higher serum uric acid can be diagnosed as hyperuricemia? (single choice)

A 300 μmol/L

B 360 μmol/L

C 420 μmol/L

D 480 μmol/L

The correct answer is C

2. Patients with gout must have hyperuricemia at a particular stage during gout. (true-false item)

A True

B False

The correct answer is A

3. Acute gout attacks must be accompanied by elevated serum uric acid levels. (true-false item)

A True

B False

The correct answer is B

4. Which are the stages of hyperuricemia? (multiple choice)

A Asymptomatic hyperuricemia

B Acute gouty arthritis

C Intermission

D Chronic gouty stone and chronic gouty arthritis

E Convalescence

The correct answers are ABCD

5. Which is the target serum uric acid level of patients with hyperuricemia less than? (single choice)

A 420 μmol/L

B 400 μmol/L

C 360 μmol/L

D 340 μmol/L

E 300 μmol/L

The correct answer is C

6. For gout patients with gout stone or chronic joint disease, which should the serum uric acid level be less than? (single choice)

A 250 μmol/L

B 300 μmol/L

C 350 μmol/L

D 400 μmol/L

E 420 μmol/L

The correct answer is B

7. Which is the lower limit of serum uric acid during uric acid-lowering therapy? (single choice)

A 100 μmol/L

B 120 μmol/L

C 140 μmol/L

D 160 μmol/L

E 180 μmol/L

The correct answer is E

8. Should early patients with hyperuricemia be treated with drugs? (true-false item)

A True

B False

The correct answer is B

9. Long-acting glucocorticoids such as dexamethasone should be avoided when acute gout attacking. (true-false item)

A True

B False

The correct answer is A

10. If the gout occurs once a year, uric acid-lowering treatments must be started. (true-false item)

A True

B False

The correct answer is B

11. For asymptomatic patients with hyperuricemia (without definite etiology or arthritis attacks), it is recommended to take non-drug treatments first and visit regularly. (true-false item)

A True

B False

The correct answer is A

12. Patients with long-term medication should regularly (3–6 months) monitor the serum uric acid level. (true-false item)

A True

B False

The correct answer is A

13. Even if serum uric acid is stable at the normal level, uric acid-lowering drugs cannot be reduced. (true-false item)

A True

B False

The correct answer is B

14. The dosage of uric acid-lowering drugs should be increased when acute gout attacking. (true-false item)

A True

B False

The correct answer is B

15. The urine pH of 6.5–6.8 is the most suitable when using sodium bicarbonate. (true-false item)

A True

B False

The correct answer is A

16. Drugs promoting uric acid excretion are often combined with sodium bicarbonate to alkalize urine

and reduce the occurrence of kidney stones. (true-false item)

A True

B False

The correct answer is A

17. Patients with gouty arthritis or acute joint symptoms, whose joint symptoms have improved by less than 50% after being treated for 24 h are regarded as having poor curative effects. (true-false item)

A True

B False

The correct answer is A

18. Which are the preferred drugs for acute gout? (multiple choice)

A Glucocorticoid

B Colchicine

C Allopurinol

D Nonsteroidal anti-inflammatory drugs

E Febuxostat

The correct answers are BD

19. Under which circumstances are patients with acute gout contraindicated to use colchicine? (multiple choice)

A Dialysis

B eGFR < 10 ml/min/1.73m^2

C eGFR 10–34 ml/min/1.73m^2

D eGFR 35–49 ml/min/1.73m^2

E eGFR 50–65 ml/min/1.73m^2

The correct answers are AB

20. Which are the drugs that inhibit uric acid synthesis? (multiple choice)

A Allopurinol

B Benzbromarone

C Febuxostat

D Colchicine

E Aspirin

The correct answers are AC

21. How much body mass index can be diagnosed as obesity? (single choice)

A 20 kg/m^2

B 22 kg/m^2

C 26 kg/m^2

D 28 kg/m^2

E 30 kg/m^2

The correct answer is D

22. Improving lifestyle is the core of treating gout and hyperuricemia. (true-false item)

A True

B False

The correct answer is A

23. For patients with hyperuricemia, it is recommended to maintain a daily water intake of more than 2 L. (true-false item)

A True

B False

The correct answer is A

24. Patients with hyperuricemia are recommended 2 eggs per day. (true-false item)

A True

B False

The correct answer is B

25. Blanching water can reduce the purine content in food. (true-false item)

A True

B False

The correct answer is A

26. Patients with hyperuricemia are encouraged to intake cereals with a low glycemic index. (true-false item)

A True

B False

The correct answer is A

27. Which are the weight management and regular exercise requirements for patients with hyperuricemia? (multiple choice)

A BMI less than 24 kg/m^2

B Male waist less than 90 cm

C Female waist less than 70 cm

D Exercise more than 5 days a week

F Moderate intensity exercise every day for more than 60 minutes

The correct answers are ABD

28. Which of the following belongs to red meat? (multiple choice)

A Fish

B Chicken

C Mutton

D Beef

E Pork

The correct answers are CDE

29. Gout can be complicated with renal failure and cardiovascular and cerebrovascular diseases and may eventually endanger life. (true-false item)

A True

B False

The correct answer is A

30. High urine pH increases the risk of calcium phosphate and calcium carbonate stones. (true-false item)

A True

B False

The correct answer is A

Part two: Questionnaire on hyperuricemia and gout among patients with diabetes

Demographic information

1. Age________years old

2. Which is your highest academic degree (including currently studying)?

A Primary school or below

B Junior high school

C High school or secondary vocational school

D Junior college

E Undergraduate college

F Master and above

3. Which is your gender?

A Male

B Female

4. Have you ever suffered from hyperuricemia?

A Yes

B No

C Unclear

5. Have you ever suffered from gout?

A Yes

B No

C Unclear

Practices and attitudes

1. Are you willing to receive training on hyperuricemia and gout?

A Yes

B No

2. Which of the following are your sources of health knowledge? (multiple choice)

A medical workers

B Relatives or friends

C Television or radio

D Books, newspapers, magazines, or other printed materials

E Network

F Lectures

G Others

Knowledge

1. Do you know hyperuricemia? (Note: if you know, continue to answer, and if not, skip to question 19)

A Know

B Unknow

2. The diagnostic criteria for hyperuricemia are serum uric acid level > 420 μmol/L.

A True

B False

C Unclear

The correct answer is A

3. Uric acid is mainly excreted from urine.

A True

B False

C Unclear

The correct answer is A

4. The diagnosis of hyperuricemia is based on urine examination.

A True

B False

C Unclear

The correct answer is B

5. The direct relatives of patients with hyperuricemia have a higher probability of suffering from the disease than healthy people.

A True

B False

C Unclear

The correct answer is A

6. Hyperuricemia is associated with gout.

A True

B False

C Unclear

The correct answer is A

7. Hyperuricemia will definitely develop into gout.

A True

B False

C Unclear

The correct answer is B

8. Hyperuricemia is associated with kidney stones.

A True

B False

C Unclear

The correct answer is A

9. Hyperuricemia can lead to a series of complications, such as hypertension, diabetes, hyperlipidemia, cerebrovascular disease, and renal failure.

A True

B False

C Unclear

The correct answer is A

10. Patients with hyperuricemia should limit animal viscera, seafood, and thick broth intake.

A True

B False

C Unclear

The correct answer is A

11. Patients with hyperuricemia should control the intake of red meat (such as pork, mutton, and beef).

A True

B False

C Unclear

The correct answer is A

12. Patients with hyperuricemia should avoid drinking sugary beverages.

A True

B False

C Unclear

The correct answer is A

13. Patients with hyperuricemia should eat more alkaline foods (such as soda).

A True

B False

C Unclear

The correct answer is A

14. Drinking more water can promote the excretion of uric acid.

A True

B False

C Unclear

The correct answer is A

15. Hyperuricemia must be treated with drugs.

A True

B False

C Unclear

The correct answer is B

16. Patients with hyperuricemia should follow the doctor's advice to take drugs regularly and reasonably and should not stop or add medicines without authorization.

A True

B False

C Unclear

The correct answer is A

17. The lower the serum uric acid level, the better.

A True

B False

C Unclear

The correct answer is B

18. Patients with hyperuricemia should regularly monitor serum uric acid levels.

A True

B False

C Unclear

The correct answer is A

19. Do you know gout? (Note: if you know, continue to answer, and if not, please end this questionnaire)

A Know

B Unknow

20. The direct relatives of gout patients have a higher probability of suffering from the disease than healthy people.

A True

B False

C Unclear

The correct answer is A

21. Gout can cause joint disease.

A True

B False

C Unclear

The correct answer is A

22. Patients with an acute gout attack or chronic gout stones can drink alcohol moderately.

A True

B False

C Unclear

The correct answer is B

23. Beer is more likely to induce gout attacks in alcohol.

A True

B False

C Unclear

The correct answer is A

24. Gout patients should avoid drinking sugary drinks.

A True

B False

C Unclear

The correct answer is A

25. Gout patients should limit animal viscera, seafood, and thick broth intake.

A True

B False

C Unclear

The correct answer is A

26. Gout patients should control the intake of red meat (such as pork, mutton, and beef).

A True

B False

C Unclear

The correct answer is A

27. When acute gouty arthritis attacks, a massage or a hot compress can be used.

A True

B False

C Unclear

The correct answer is B

28. Patients with gout should take X-ray films regularly to observe joint lesions.

A True

B False

C Unclear

The correct answer is A

Table S1. Knowledge rate of hyperuricemia and gout among community health workers

Number	Item	Accuracy % (n)			
		GPs	Nurses	Others	Total
**Pathophysiology**					
A1	How much higher SUA can be diagnosed as hyperuricemia?	72.30% (201)	43.89% (140)	51.79% (58)	56.28% (399)
A2	Patients with gout must have hyperuricemia at a particular stage during gout.	80.22% (223)	93.10% (297)	92.86% (104)	88.01% (624)
A3	Acute gout attacks must be accompanied by elevated serum uric acid levels.	64.75% (180)	20.38% (65)	23.21% (26)	38.22% (271)
A4	Which are the stages of hyperuricemia?	23.74% (66)	11.6% (37)	13.39% (15)	16.64% (118)
Average accuracy		60.25%	42.24%	45.31%	49.79%
**Drug treatments**					
B1	Which is the target SUA level of patients with hyperuricemia less than?	52.88% (147)	33.86% (108)	38.39% (43)	42.03% (298)
B2	For gout patients with gout stone or chronic joint disease, which should the SUA level be less than?	52.16% (145)	35.11% (112)	27.68% (31)	40.62% (288)
B3	Which is the lower limit of serum uric acid during uric acid-lowering therapy?	44.96% (125)	21.32% (68)	33.04% (37)	32.44% (230)
B4	Should early patients with hyperuricemia be treated with drugs?	82.01% (228)	34.48% (110)	44.64% (50)	54.72% (388)
B5	Long-acting glucocorticoids such as dexamethasone should be avoided when acute gout attacking.	85.61% (238)	89.34% (285)	92.86% (104)	88.43% (627)
B6	If the gout occurs once a year, uric acid-lowering treatments must be started.	19.78% (55)	11.91% (38)	16.96% (19)	15.80% (112)
B7	For asymptomatic patients with hyperuricemia (without definite etiology or arthritis attacks), it is recommended to take non-drug treatments first and visit regularly.	88.49% (246)	91.85% (293)	93.75% (105)	90.83% (644)
B8	Patients with long-term medication should regularly (3–6 months) monitor the SUA level.	98.92% (275)	94.98% (303)	96.43% (108)	96.76% (686)
B9	Even if SUA is stable at the normal level, uric acid-lowering drugs cannot be reduced.	57.19% (159)	23.20% (74)	31.25% (35)	37.80% (268)
B10	The dosage of uric acid-lowering drugs should be increased when acute gout attacking.	61.51% (171)	27.90% (89)	21.43% (24)	40.06% (284)
B11	The urine pH of 6.5–6.8 is the most	90.65%	87.15%	88.39%	88.72%

	suitable when using sodium bicarbonate.	(252)	(278)	(99)	(629)
B12	Drugs promoting uric acid excretion are often combined with sodium bicarbonate to alkalize urine and reduce the occurrence of kidney stones.	97.84% (272)	96.24% (307)	95.54% (107)	96.76% (686)
B13	Patients with gouty arthritis or acute joint symptoms, whose joint symptoms have improved by less than 50% after being treated for 24 h are regarded as having poor curative effects.	73.74% (205)	83.39% (266)	84.82% (95)	79.83% (566)
B14	Which are the preferred drugs for acute gout?	11.15% (31)	1.25% (4)	2.68% (3)	5.36% (38)
B15	Under which circumstances are patients with acute gout contraindicated to use colchicine?	13.31% (37)	5.33% (17)	3.57% (4)	8.18% (58)
B16	Which are the drugs that inhibit uric acid synthesis?	34.89% (97)	5.64% (18)	14.29% (16)	18.48% (131)
Average accuracy		60.32%	46.43%	49.11%	52.30%
**Lifestyle interventions**					
C1	How much body mass index can be diagnosed as obesity?	65.83% (183)	56.43% (180)	39.29% (44)	57.40% (407)
C2	Improving lifestyle is the core of treating gout and hyperuricemia.	97.84% (272)	99.06% (316)	94.64% (106)	97.88% (694)
C3	For patients with hyperuricemia, it is recommended to maintain a daily water intake of more than 2 L.	96.76% (269)	95.92% (306)	98.21% (110)	96.61% (685)
C4	Patients with hyperuricemia are recommended 2 eggs per day.	79.50% (221)	58.93% (188)	56.25% (63)	66.57% (472)
C5	Blanching water can reduce the purine content in food.	82.01% (228)	82.13% (262)	83.93% (94)	82.37% (584)
C6	Patients with hyperuricemia are encouraged to intake cereals with a low glycemic index.	95.32% (265)	87.46% (279)	91.07% (102)	91.11% (646)
C7	Which are the weight management and regular exercise requirements for patients with hyperuricemia?	11.51% (32)	2.82% (9)	0.89% (1)	5.92% (42)
C8	Which of the following belongs to red meat?	61.87% (172)	34.48% (110)	45.54% (51)	46.97% (333)
Average accuracy		73.87%	64.65%	63.73%	68.11%
**Harmful effects**					
D1	Gout can be complicated with renal failure and cardiovascular and cerebrovascular diseases and may eventually endanger life.	100% (278)	97.81% (312)	96.43% (108)	98.45% (698)

D2	High urine pH increases the risk of calcium phosphate and calcium carbonate stones.	92.09% (256)	96.87% (309)	97.32% (109)	95.06% (674)
Average accuracy		96.05%	97.34%	96.88%	96.76%
**Total**					
Average accuracy		66.31%	54.12%	55.69%	59.15%

Abbreviation: GPs, general practitioners; SUA, serum uric acid.

Table S2. Knowledge rate of hyperuricemia and gout among patients with diabetes

Numbers	Items	Accuracy % (n)	Number of respondents
**Pathophysiology**			
A1	The diagnostic criteria for hyperuricemia are SUA level > 420 μmol/L.	37.80% (62)	164
A2	Uric acid is mainly excreted from urine.	75.00% (123)	164
A3	The diagnosis of hyperuricemia is based on urine examination.	37.20% (61)	164
A4	Hyperuricemia is hereditary	29.88% (49)	164
A5	Gout is hereditary	39.81% (123)	309
Average accuracy		43.94%	-
**Harmful effects**			
B1	Hyperuricemia is associated with gout.	96.95% (159)	164
B2	Hyperuricemia will definitely develop into gout.	12.20% (20)	164
B3	Hyperuricemia is associated with kidney stones.	50.00% (82)	164
B4	Hyperuricemia can lead to a series of complications such as hypertension, diabetes, hyperlipidemia, cerebrovascular disease, renal failure.	63.41% (104)	164
B5	Gout can cause joint disease.	84.14% (260)	309
Average accuracy		61.34%	-
**Treatments**			
C1	Patients with hyperuricemia should limit animal viscera, seafood, and thick broth intake.	95.12% (156)	164
C2	Patients with hyperuricemia should control the intake of red meat (such as pork, mutton, and beef).	72.56% (119)	164
C3	Patients with hyperuricemia should avoid drinking sugary beverages.	72.56% (119)	164

C4	Patients with hyperuricemia should eat more alkaline foods (such as soda).	76.83% (126)	164
C5	Drinking more water can promote the excretion of uric acid.	92.68% (152)	164
C6	Hyperuricemia must be treated with drugs.	28.66% (47)	164
C7	Patients with hyperuricemia should follow the doctor’s advice to take drugs regularly and reasonably and should not stop or add medicines without authorization.	90.24% (148)	164
C8	The lower the SUA level, the better.	51.22% (84)	164
C9	Patients with hyperuricemia should regularly monitor SUA levels.	92.07% (151)	164
C10	Patients with an acute gout attack or chronic gout stones can drink alcohol moderately.	63.43% (196)	309
C11	Beer is more likely to induce gout attacks in alcohol.	92.56% (286)	309
C12	Gout patients should avoid drinking sugary drinks.	66.02% (204)	309
C13	Gout patients should limit animal viscera, seafood, and thick broth intake.	92.56% (286)	309
C14	Gout patients should control their intake of red meat (such as pork, mutton, and beef).	67.64% (209)	309
C15	When acute gouty arthritis attacks, a massage or a hot compress can be used.	33.33% (103)	309
C16	Patients with gout should take X-ray films regularly to observe joint lesions.	75.73% (234)	309
Average accuracy		72.70%	-
**Total**			
Average accuracy		64.98%	-

Abbreviation: SUA, serum uric acid.

Table S3. Multiple linear regression on factors associated with knowledge scores in community health workers

Variable	SE	*P*-value	95% CI for B	VIF
Male (*vs.* female)	0.346	<0.001*	-1.471[-2.149, -0.792]	1.116
**Occupations**				
GPs (*vs.* Nurses)	0.280	<0.001*	3.794 [3.244, 4.344]	1.493
Others (*vs.* Nurses)	0.338	0.028*	0.743 [0.080, 1.406]	1.212
**Age (years)**				
< 30 (*vs.* 30-39)	0.273	0.150	-0.394 [-0.930, 0.142]	1.183
40–49 (*vs.* 30-39)	0.344	0.827	0.075 [-0.601, 0.751]	1.155
≥ 50 (*vs.* 30-39)	0.661	0.675	-0.277[-1.576, 1.021]	1.279

**Educational levels**				
Secondary vocational school education (*vs.* Bachelor's degree)	0.748	0.068	-1.366 [-2.835, 0.103]	1.225
Junior college degree (*vs.* Bachelor's degree)	0.257	0.596	-0.136 [-0.640, 0.368]	1.197
Master's degree (*vs.* Bachelor's degree)	0.746	0.699	0.288 [-1.177, 1.754]	1.041

Abbreviation: SE, standard error; CI, confidence interval; VIF, variance inflation factor; GPs, general practitioners; *$P \leq 0.05$.

Table S4. Multiple linear regression on factors associated with knowledge scores in patients with diabetes

Variable	SE	*P*-value	95% CI for B	VIF
Male (*vs.* female)	0.643	0.593	0.344 [-0.919, 1.606]	1.081
History of hyperuricemia or gout (*vs.* no)	0.669	<0.001*	3.792 [2.478, 5.106]	1.029
**Age (years)**				
< 50 (*vs.* 50-69)	1.540	0.202	1.969 [-1.058, 4.995]	1.076
≥ 70 (*vs.* 50-69)	0.670	0.067	-1.228 [-2.545, 0.088]	1.098
**Educational levels**				
Junior high school (*vs.* primary school or below)	0.734	<0.001*	3.657 [2.214, 5.100]	1.301
High school or secondary vocational school (*vs.* primary school or below)	0.902	<0.001*	3.726 [1.954, 5.499]	1.278
Junior college or undergraduate college (*vs.* primary school or below)	1.412	<0.001*	7.434 [4.661, 10.208]	1.162

Abbreviation: SE, standard error; CI, confidence interval; VIF, variance inflation factor; *$P \leq 0.05$.
